# A Lecture, Introductory to a Course of Clinical Medicine, Delivered in the Theatre of Queen's College, Birmingham, &c. &c.

**Published:** 1847-07

**Authors:** 


ART. IV.—*A Lecture, introductory to a Course of Clinical Medicine, delivered in the Theatre of Queen's College, Birmingham, &c. &c.* By SAMUEL WRIGHT, M.D., &c.

AN interesting brochure, and containing sound truths well expressed. We doubt not, that as Professor of Clinical Medicine, Dr. Wright will bring his talents and energy to bear upon his duties, and the profession as well as his class will benefit by his labours.

